# Epidemiological profile of leprosy cases at Nazareti Leprosy Center, Tanzania: A retrospective analysis (2000–2024)

**DOI:** 10.1371/journal.pntd.0014761

**Published:** 2026-09-30

**Authors:** Robert Machang’u, Jacob N. Kagesyeko, Majani Edward, Wisdom Owoicho Ameh, Lucy Chuwa, Gabriel Upunda, Jovin R. Tibenderana

**Affiliations:** 1 Department of Microbiology and Parasitology, St. Francis University College of Health and Allied Sciences, Ifakara, Morogoro, Tanzania; 2 Research and Training Unit, Department of Research and Innovation, MacWish College of Health and Allied Sciences, Mwanza, Tanzania; 3 Centre for Reforms, Innovation, Health policies and Implementation Research, Dodoma, Tanzania; 4 Federal University of Technology, Minna, Nigeria; 5 Nazareti Leprosy Center, Ifakara, Morogoro, Tanzania; 6 Department of Internal Medicine and Clinical Pharmacology, St. Francis University College of Health and Allied Sciences, Ifakara, Morogoro, Tanzania; 7 Department of Public Health, St. Francis University College of Health and Allied Sciences, Ifakara, Morogoro, Tanzania; University of Florida College of Medicine, UNITED STATES OF AMERICA

## Abstract

**Introduction:**

Leprosy, also commonly known as Hansen’s Disease (HD) remains a neglected tropical disease and a public health concern in disease endemic regions of the world. However, significant progress in disease reduction has been achieved in recent years through multidrug therapy programs, implemented by the World Health Organization (WHO). This study assessed the trend and epidemiological profile of HD cases diagnosed at Nazareti Leprosy Center (NLC), Ifakara, Tanzania, over a 25-year period.

**Methods:**

A retrospective cross-sectional study was conducted using routinely collected clinical records of newly diagnosed HD cases registered at NLC between 2000 and 2024. Data on demographic characteristics, clinical classification of HD, operational categories (paucibacillary and multibacillary), and disability grades of patients at presentation were collected. Poisson regression estimated annual incidence trends as incidence rate ratios (IRRs), while logistic regression assessed changes in leprosy characteristics as odds ratios (ORs), both reported with 95% confidence intervals (CIs).

**Results:**

A total of 801 HD cases were recorded, of which males accounted for 67.4% of cases, with the highest proportion occurring among individuals aged 50 + years (33.2%). Multibacillary disease predominated (62.7%), while grade 1 disability (G1D), at diagnosis was more common (52.7%) than G2D. Overall, case notifications increased significantly over time, with an average annual rise of 4.0% (IRR = 1.04; 95% CI: 1.03–1.07; p < 0.001). Significant increases were observed among male individuals aged ≥50 years. Multibacillary HD cases and G1D disability also increased. In contrast, childhood cases (0–14 years) declined significantly during the study period.

**Conclusions:**

Leprosy epidemiology in rural Tanzania shows evolving patterns characterized by increasing case detection, a rising burden of multibacillary disease, and disability at diagnosis, versus a decline in childhood cases over time. Intensifying early case detection, community awareness, treatment and surveillance are essential to eliminate HD as a public health concern.

## Author summary

Although global efforts have reduced the burden of Hansen Disease (leprosy), it was found that the number of people diagnosed at Nazareti Leprosy Center (in rural Tanzania) has steadily increased over time. Most cases occurred in older adults and men, and a large proportion of patients had more advanced forms of the disease at the time of diagnosis. It was also observed that a number of patients had early signs of disability when they first came for care, suggesting delays in seeking or receiving treatment. At the same time, records showed a decline in cases among children, which may indicate some progress in reducing recent transmission. Taken together, the findings show that while control efforts may be having some effect, important challenges remain, particularly delays in diagnosis and ongoing transmission in the community. Enhancing community awareness, encouraging early care-seeking and strengthening local health services are essential steps toward reducing disability and eventually eliminating this disease as a public health problem.

## Background

Leprosy, also known as Hansen’s disease, is a chronic bacterial disease caused by *Mycobacterium leprae*, a Gram-negative bacillus, which affects the dermis and peripheral nerves and can also involve the eye, the mucosa of the upper respiratory tract, muscle, bone, and testes [1]. If treatment is not provided timely, the infection may result in progressive nerve damage and irreversible disability [2]. While the introduction of multidrug therapy (MDT) in the 1980s was a major milestone, modern leprosy control has evolved into a comprehensive strategy. Current global and national frameworks now emphasize targeted elimination campaigns in endemic areas, active case finding, systematic contact tracing, post-exposure prophylaxis (PEP), and dedicated disability prevention initiatives [3]. Despite these multifaceted interventions, leprosy remains an important public health concern in many low- and middle-income countries due to persistent community transmission, delayed diagnosis, stigma, and social exclusion [4].

Globally, leprosy detection shows mixed trends with 182,815 new cases reported in 2023, decreasing slightly to 172,717 cases in 2024, corresponding to a new case detection rate of 21.1 per million population [5]. Despite this decline, critical epidemiological indicators reveal persistent transmission and delayed diagnosis. In 2024, multibacillary (MB) cases continued to dominate, representing over 70% of new detections globally, childhood cases (<15 years) accounted for 5.4% (9,397 children) of total cases, and 5.3% (9,157 patients) presented with Grade 2 disability (visible deformities) at diagnosis [5]. Among children diagnosed, 3.1% already had Grade 2 disability, indicating severe delays in case detection among the youngest and most vulnerable population, these figures have remained largely stagnant over the past decade, with the proportion of child cases persisting at 5–6% and Grade 2 disability rates hovering around 5%, demonstrating that global progress toward interruption of transmission has plateaued [5].

Globally, leprosy detection rates have remained relatively stable over the past decade, with approximately 200,000 new cases reported annually. The highest burden is concentrated in three countries: India reported 100,957 new cases in 2024, Brazil reported 22,773 new cases in 2023, and Indonesia reported 14,698 new cases in 2024 [6–8], together, these three countries account for approximately 79% of all new leprosy cases detected globally [9]. In 2023, the South-East Asia and African regions reported the highest burden of new case detection globally, with the South-East Asia Region alone contributing 71.9% (131,425 cases) of all new cases, followed by the African Region contributing 11.5% (21,043 cases) [7,10].

Tanzania is listed among the 25 highest leprosy endemic countries, with 1,454 new cases reported in 2023 [11]. The national strategy focuses on reducing the incidence of Grade 2 disabilities and eliminating childhood leprosy, which are key indicators of the program’s success in interrupting transmission. However, modeling studies estimate that over 2.6 million leprosy cases remain undiagnosed globally, representing a significant hidden reservoir of transmission [12], In Tanzania, this underreporting is compounded by severe diagnostic delays, with recent studies reporting a mean case detection delay of 28.1 months, indicating that official notified figures substantially underrepresent the true community burden of the disease [13]

Nationally, key epidemiological markers indicate persistent transmission and delayed diagnosis. For instance, multibacillary (MB) cases consistently constitute 92.9% of new detections, reflecting a high bacterial load and prolonged community transmission [14]. Furthermore, the proportion of patients presenting with Grade 2 disability at diagnosis has remained stagnant at approximately 10% recorded in both 2020 (1,208 new cases) and 2021 (1,511 new cases) which is nearly double the global average and highlights systemic delays in case detection [14].

National surveillance data often masks localized transmission dynamics and long-term shifts in disease severity at the facility level. Specifically, there is a critical lack of long-term, granular data evaluating how the proportions of high-risk epidemiological indicators have evolved over decades in Tanzania’s leprosy centers. To address this gap, this study aimed to assess the temporal trends and epidemiological profile of HD cases diagnosed at NLC, Ifakara, over a 25-year period (2000–2024)

The findings will provide actionable, facility-level evidence to refine targeted active-case-finding and disability prevention strategies, ultimately supporting Tanzania’s progress toward leprosy elimination. Findings will also provide insights into changes in the disease burden and patient characteristics over time, generating evidence to inform national leprosy control strategies, strengthening early case detection, reducing disability and supporting progress towards leprosy elimination in the country

## Methodology

### Ethics statement

Approval to conduct the study was obtained from NLC Management,and Ethical Clearance was obtained from St. Francis University College of Health and Allied Sciences (SFUCHAS)-Institutional Review Board (IRB). Since the study relied on retrospective review of medical records, no direct patient contact was involved and confidentiality of patient information was maintained throughout the study. Personal identifiers were not collected during data extraction, and all information obtained from the records was used strictly for this research purposes only

### Study design

A retrospective cross-sectional study design was employed to review routinely collected patient records from the Center register.

### Study area and population

The study was conducted at NLC, historically a Roman Catholic Church charity establishment -“refugial home”- for ostracized lepers”. Since its founding, in 1964, NLC has transformed into a dependable referral Center, where leprosy diagnosis and treatment are provided free of charge. The NLC is now linked with the Ifakara District Tuberculosis and Leprosy Centre, and the National TB and Leprosy Program Office in Ifakara Town Council. The larger Morogoro Region, to which the Ifakara Town Council forms part, covers approximately 70,624 km^2^ and had an estimated population of 3,197,104 [15]. The Region comprises nine administrative District Councils, including the Kilombero Council, where Ifakara is the major town. NLC primarily serves patients from Ifakara, the neighbouring districts and, to a lesser extent, from elsewhere in Tanzania. As a referral facility, therefore, the Center does not have a strictly defined catchment population. The homes of residence and number of patients attended are therefore, variably distributed geographically.

The study population consisted of all individuals diagnosed with HD and registered for care at NLC during the 2000–2024 period. Information was obtained from hard copies of patient medical records (files) maintained at the Center.

### Data source and management

Patients are registered after confirmation of diagnosis, and demographic and clinical information is documented in the Facility registers. These records served as the main source of data for the study.

Patient information was recorded by trained clinical officers, using standardized national leprosy reporting tools based on Tanzania National Leprosy and Tuberculosis Programme and the World Health Organization guidelines. Data extracted included year of diagnosis, age, sex, place of residence, WHO operational classification (PB/MB), disability grade at diagnosis, treatment initiation, and household contact history where available. A standardized data extraction form was used, and the data were entered into a password-protected electronic excel database after checking for completeness, consistency, and accuracy against the original records. Although minor modifications to the registers may have occurred over the 25-year period, the core surveillance variables remained consistently recorded using standardized procedures

### Data collection

Data were extracted using a paper based structured data extraction form specifically developed for the study. Information collected included: Sociodemographic characteristics, such as age and sex, clinical characteristics, including type of leprosy and disability status at diagnosis, year of diagnosis, treatment initiation.

### Case classification

Cases were classified, according to the WHO operational grading system, as PB or MB based on the number of skin lesions and clinical findings recorded in the patient files. Paucibacillary leprosy generally referred to cases presenting with a “few” number (< 6) of skin lesions without detectable bacilli by microscopy, while multibacillary (MB) leprosy included cases with more extensive lesions or confirmed bacterial presence by the slit skin smear method.

### Operational definitions

**Paucibacillary (PB) leprosy**: Leprosy with 1–5 skin lesions and no detectable bacilli on slit-skin smear, according to the World Health Organization operational classification.

**Multibacillary (MB) leprosy**: Leprosy with more than five skin lesions or a positive slit-skin smear for Mycobacterium leprae, according to the World Health Organization operational classification.

**Grade 0 Disability (G0D)**: No loss of sensation and no visible deformity or damage to the eyes, hands, or feet at diagnosis.

**Grade 1 Disability (G1D)**: Loss of protective sensation in the eyes, hands, or feet without visible deformity or damage.

**Grade 2 Disability (G2D)**: Visible deformity or damage of the eyes, hands, or feet, including ulcers, claw hand, foot drop, lagophthalmos, or severe visual impairment, present at the time of diagnosis.

### Study variables

The primary outcome variable was the number of newly diagnosed leprosy cases recorded at NLC from 2000 to 2024. Other outcome variables included: WHO operational classification (paucibacillary or multibacillary), disability grade at diagnosis (Grade 0, Grade 1, or Grade 2), age group, and sex. The primary independent variable was year of diagnosis, treated as a continuous variable to assess temporal trends in the epidemiological indicators.

### Data analysis

Collected data was entered and analyzed using STATA v.18.0, and descriptive statistics were used to summarize the characteristics of the study population. Complete case analysis was done to account for missing data. Categorical variables such as age, sex, HD type and disability status were presented as frequencies and percentages. Graphical presentations were also generated to illustrate patterns and trends in HD cases over time. To evaluate changes in the number of HD cases over time, Poisson regression analysis was applied to estimate the annual variation in incidence and to calculate the incidence rate ratio (IRR) for overall case trends, goodness-of-fit of the Poisson regression model was assessed using deviance and Pearson goodness-of-fit statistics. The model was considered appropriate when no substantial evidence of overdispersion or poor fit was observed. In addition, separate binary logistic regression models were fitted with year of diagnosis as the independent variable and the following dependent variables: childhood leprosy (0–14 years vs. ≥ 15 years), sex (male vs. female), WHO classification (multibacillary vs. paucibacillary), and disability status (Grade 1 disability vs. other disability grades). Variables were selected *a priori* based on their epidemiological importance in leprosy surveillance. Results from Poisson regression were reported as Incidence Rate Ratios (IRRs) with 95% confidence intervals (CIs), while logistic regression results were reported as Odds Ratios (ORs) with 95% CIs. Statistical significance was considered at *p* < 0.05.

## Results

A total of 801 HD cases were recorded at NLC between 2000 and 2024 (Table 1). The study population demonstrated a marked male preponderance, with 540(67.4%) cases. The age distribution revealed the highest concentration of cases in the 50 + years age group (n = 266, 33.2%). Multibacillary (MB) leprosy predominated, representing 502 cases (62.7%). Regarding disability status at diagnosis, grade 1 disability (G1D) was most prevalent (n = 421, 52.7%). Geographically, cases were distributed across multiple administrative divisions, with more than one-third of the study population originating from residential locations in the Ifakara division (n = 301, 37.6%)

**Table 1:** Demographic characteristics of leprosy cases attended at Nazareti Leprosy Center 2000-2024(N = 801).

Variable	Frequency	Percentage (%)
**Age**		
0-14	45	5.6
15-24	99	12.4
25-34	168	21.0
35-49	223	27.8
50+	266	33.2
**Sex**		
Male	540	67.4
Female	261	32.6
Leprosy classification*		
MB	502	62.7
PB	293	36.7
NA	6	0.6
Disability grade*		
G0D	315	40.7
G1D	421	52.7
G2D	53	6.6
**Location of residence**		
Ifakara	301	37.6
Kisaki	20	2.5
Mang’ula	70	8.7
Mbingu	34	4.2
Mngeta	33	4.1
Msolwa	17	2.1
Other	326	40.7

*Frequencies (n) do not tally to the total due to missing values in these variables.

MB-Multibacillary; PB- Paucibacillary; NA-Not Available classification in the data.

### Trends of leprosy diagnosis at Nazareti Leprosy Center

The overall number of HD cases attending the Nazareti Leprosy Center demonstrated a significant upward trajectory over the 25-year period (Fig 1), with an average annual increase of 4.0% (IRR = 1.04, 95% CI: 1.03–1.07, p < 0.001).

**Fig 1:**
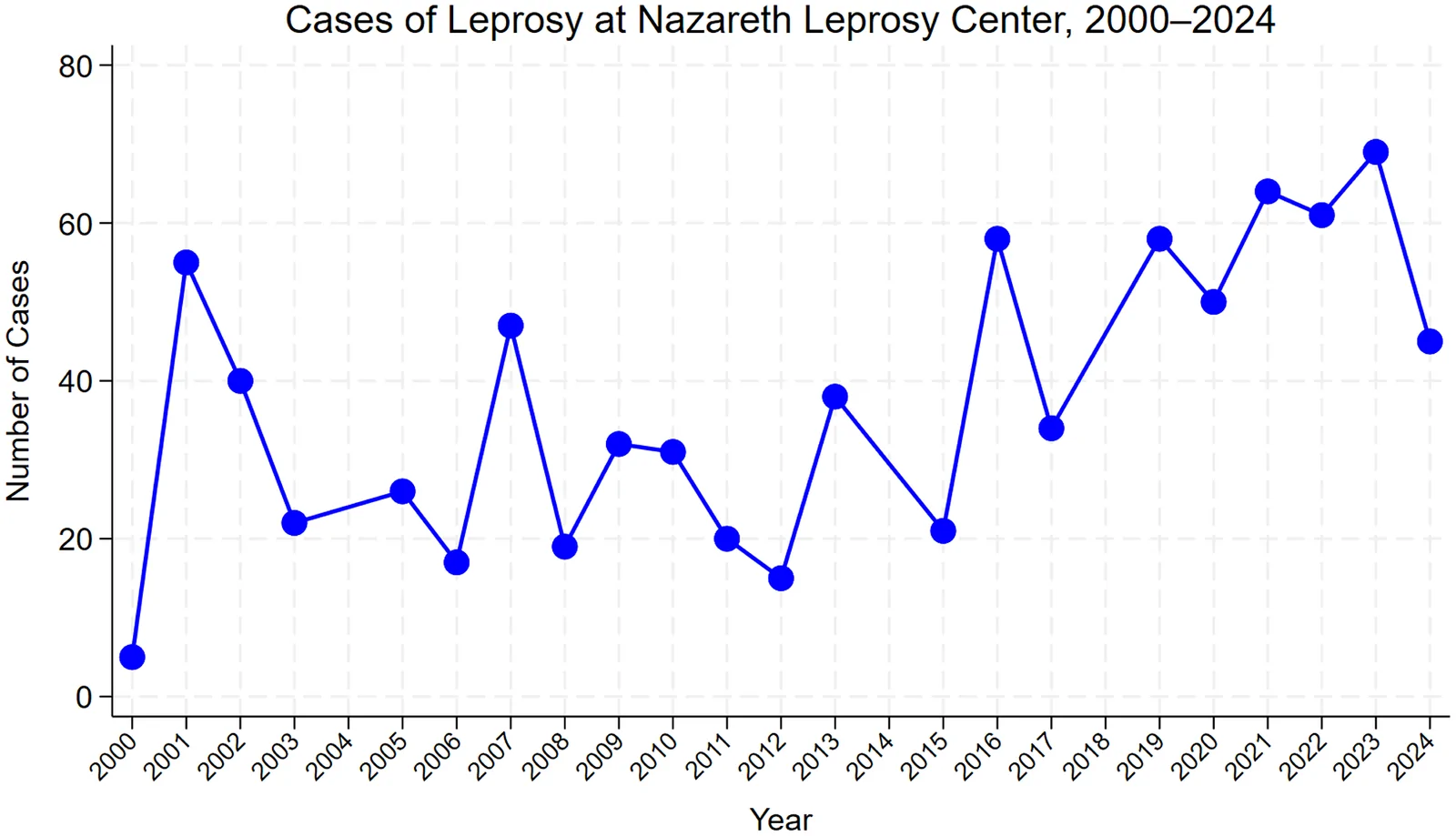
Trends of leprosy cases attended at Nazareti Leprosy Center.

### Temporal trends in key epidemiological indicators

Analysis of demographic and clinical indicators revealed several significant temporal shifts in HD case profile (Figs 2–5 and Table 2). **Table 2** illustrates the temporal trends in leprosy indicators at the Nazareti Leprosy Center from 2000 to 2024. The odds of older adults (aged 50 years and above) cases increased substantially over the study period, with an annual increase of approximately 7.0%. (OR=1.07, 95% CI: 1.05–1.09, p < 0.001). Similarly, the odds of male cases increased by approximately 5.0% annually (OR=1.05, 95% CI: 1.03–1.06, p < 0.001).

**Table 2:** Statistical tests for temporal trends in leprosy indicators at the Nazareti Leprosy Center from 2000 to 2024.

Indicator	Annual Change (OR)	95% CI	P-value
Aged (50 + Years) cases	1.07	1.05-1.09	<0.001
Male cases	1.05	1.03-1.06	<0.001
Multibacillary cases	1.05	1.03-1.06	<0.001
Child (0–14Years) cases	0.94	0.91-0.98	0.009
Grade 1 disability	1.19	1.16-1.21	<0.001

**Fig 2:**
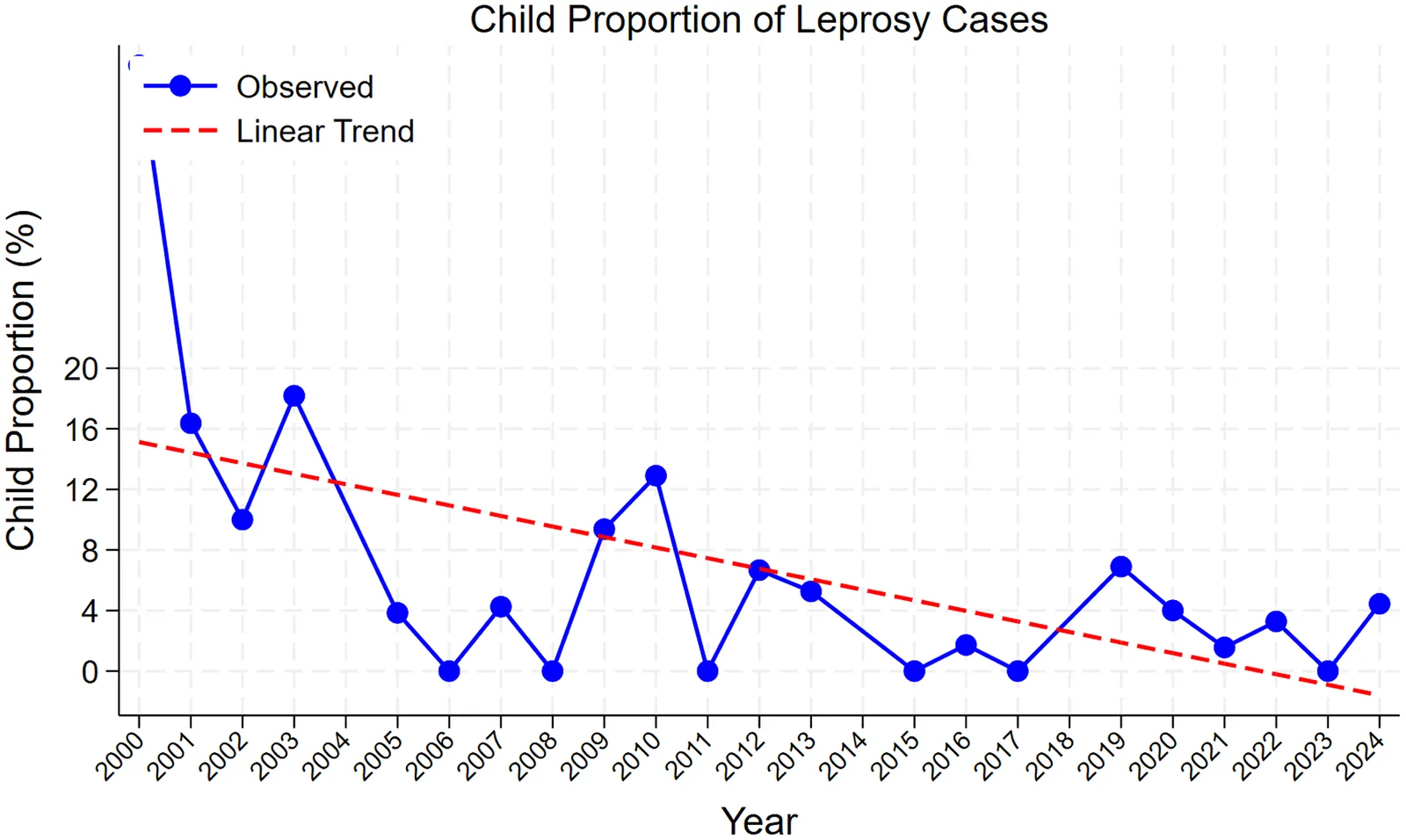
Trends of leprosy cases in children at Nazareti Leprosy Center.

**Fig 3:**
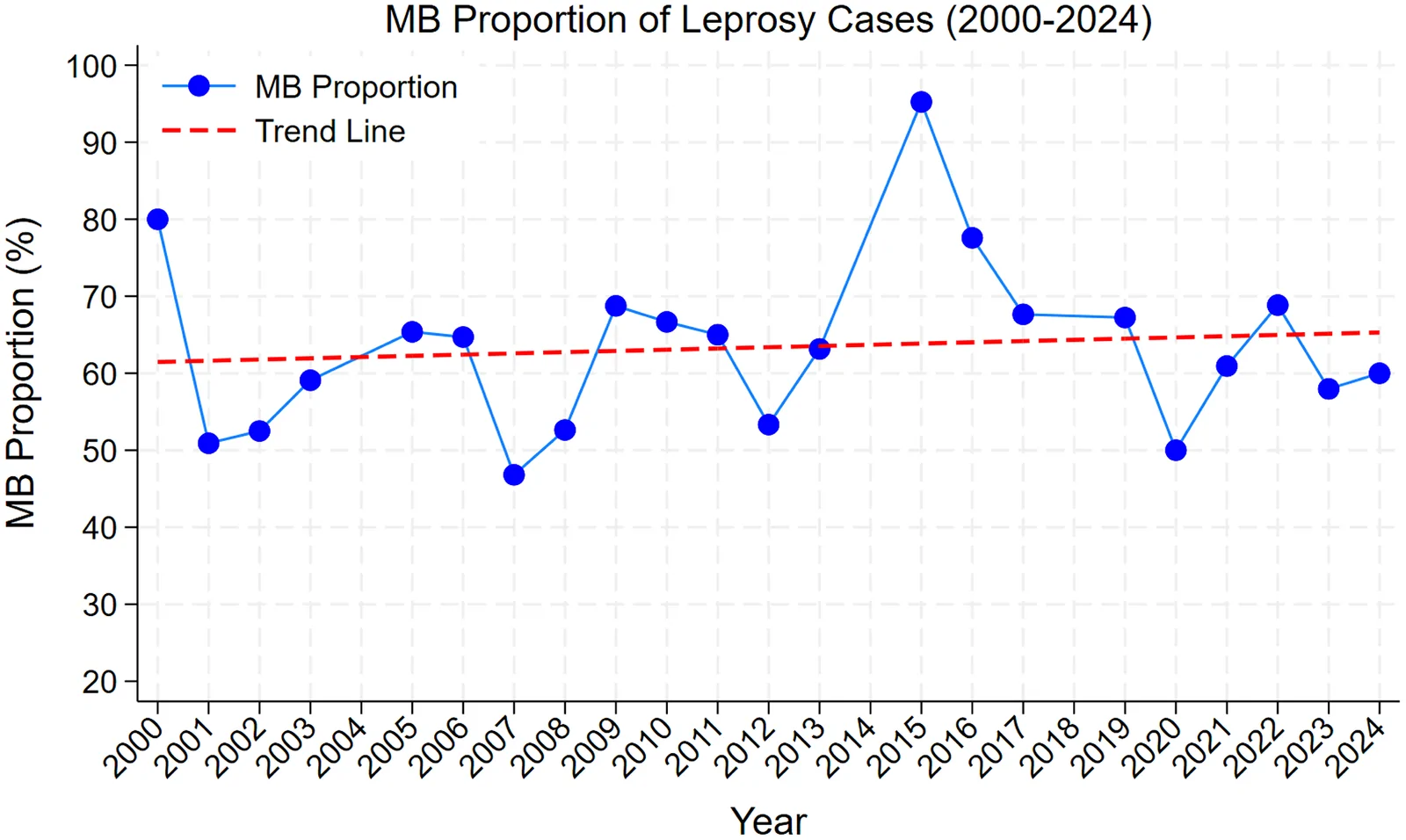
Trends of multibacillary leprosy at Nazareti Leprosy Center.

**Fig 4:**
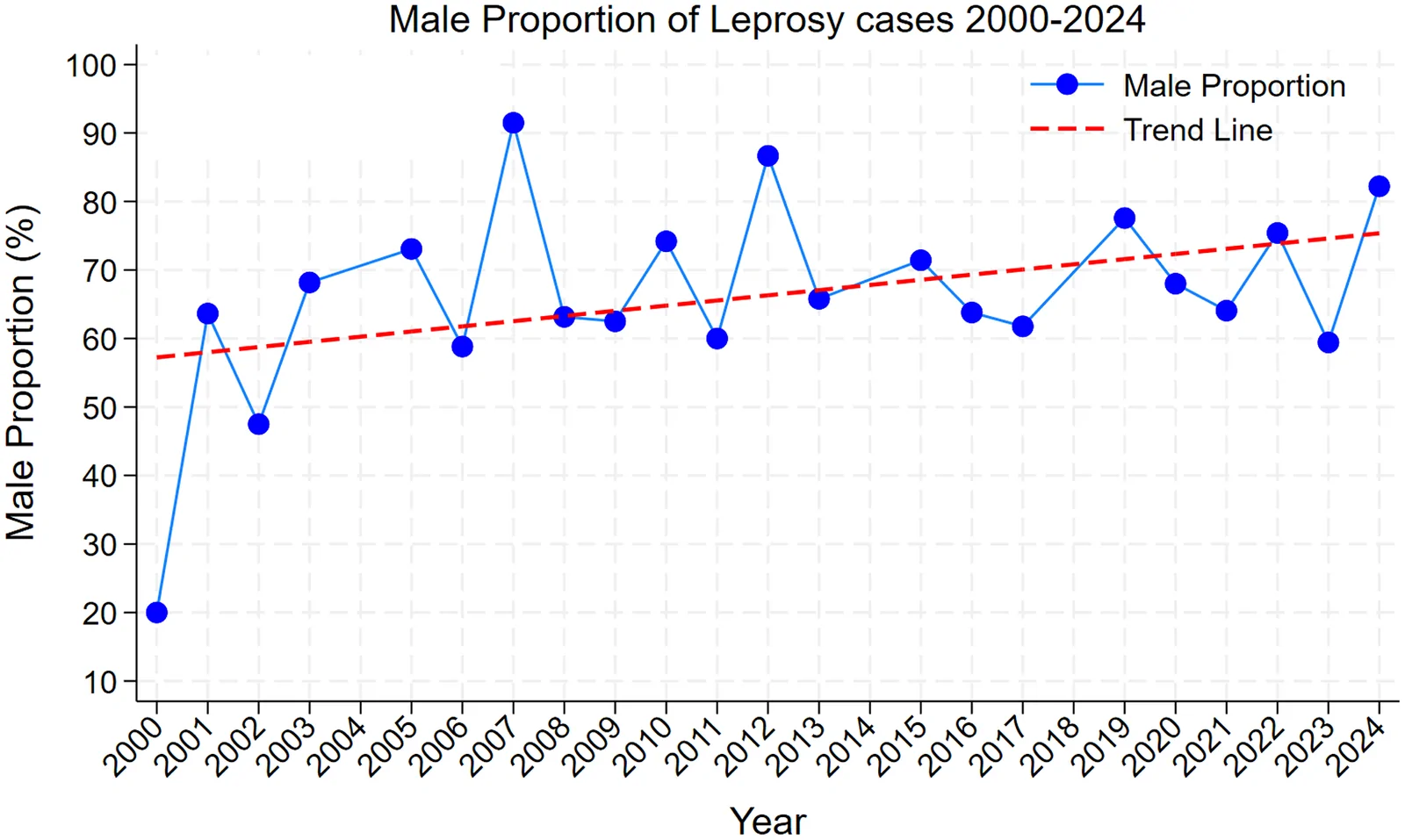
Trends of leprosy in males at Nazareti Leprosy Center.

**Fig 5:**
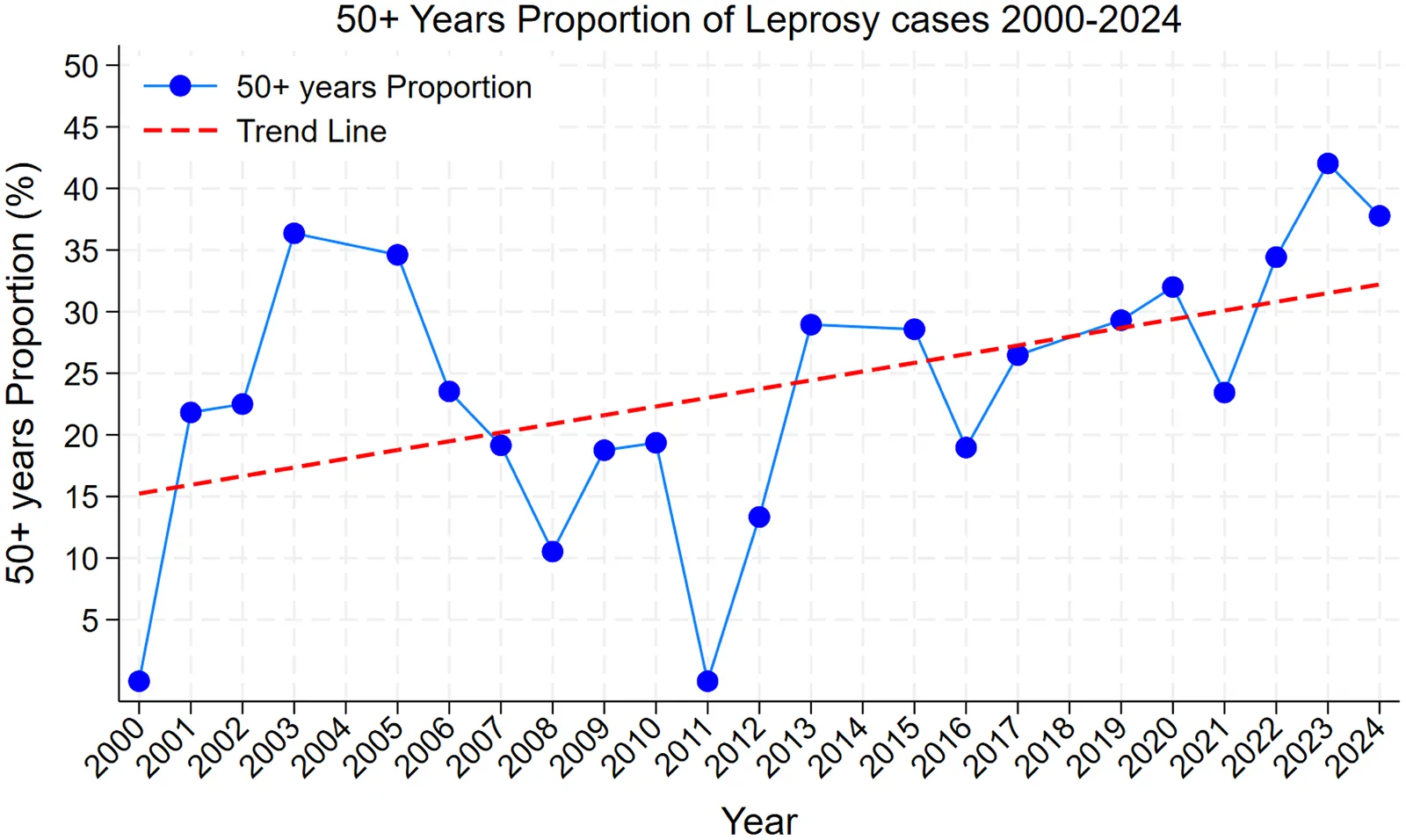
Trends of leprosy in 50+ years adults at Nazareti Leprosy Center.

The odds of multibacillary (MB) cases also increased by approximately 5.0% annually over the study period (OR=1.05, 95% CI: 1.03–1.06, p < 0.001). The odds of G1D increased by approximately 19% for each additional year (OR=1.19, 95% CI: 1.16–1.21, p < 0.001) (Fig 4).

In contrast, child cases (aged 0–14 years) demonstrated a significant decline over the 25 year period, the odds of child cases showed a 6.0% annual reduction (OR=0.94, 95% CI: 0.91–0.98, p = 0.009) (Fig 2).

## Discussion

This study examined long-term epidemiological trends of HD cases diagnosed at NLC over a 25-year period. The findings reveal several notable shifts in the epidemiological profile of HD in the study setting. Overall, the number of reported cases increased significantly over time, accompanied by a growing proportion of MB disease, similarly there was increasing representation of older adult males, and a substantial rise in G1D at diagnosis. Conversely, cases among children declined significantly. These trends collectively provide valuable insight into the evolving epidemiology and the potential challenges facing control efforts in endemic settings.

The increasing number of diagnosed cases observed in this study contrasts with the broader global decline reported over the past several decades, following the introduction of MDT [16,17]. Nevertheless, more than 200,000 new HD cases continue to be reported annually, worldwide, indicating that transmission persists in several endemic regions [18,19]. Similar upward trends in case detection have been documented in certain sub-national settings in countries such as Brazil, India, and Indonesia, often attributed to strengthened surveillance systems, or the expansion of specialized diagnostic services [13,14]. The rise in case notifications may, therefore, reflect increased awareness and health service utilization at NLC, enhanced diagnostic capacity, or ongoing transmission within surrounding communities. However, the sustained increase over a prolonged period also reveals that HD is still an important public health concern in Ifakara division, and possibly elsewhere in Tanzania.

A key finding of this study was the predominance and increasing trend of MB cases, which accounted for more than sixty percent of all cases, and demonstrated a statistically significant upward trajectory over the study period. Similar findings have been reported in other leprosy-endemic settings and are commonly associated with delayed diagnosis and prolonged disease progression before treatment initiation [20,21]. Studies from Brazil and India have, likewise, documented increasing proportions of MB cases among detected patients, suggesting persistent challenges in early case detection [22,23]. The increase of MB-HD, observed in this study, may be a reflection of delayed diagnosis, delayed inception of treatment, and may also indicate persistent transmission within the community. The demographic profile of patients also reflected striking HD shifts over time. Whereas males represented the majority of cases overall, the proportion of female cases decreased steadily during the study period. Globally, leprosy has been reported more frequently among males, with male-to-female ratios ranging from 1.5:1–2:1 in endemic regions [24,25]. The male predominance observed in this study may reflect contextual factors specific to the study setting, including greater occupational and environmental exposure, gender-related differences in health-seeking behavior, or targeted community outreach activities. The increasing trend of HD among males observed in this study is consistent with findings reported in other endemic settings [24,25].

Another notable epidemiological shift was the higher proportion of cases among individuals aged 50 years and above. Hansen’s disease is characterized by a long incubation period of several years, or even decades. Consequently, infections acquired earlier in life may first become clinically apparent in adulthood. Similar demographic transitions toward older age groups have been reported in countries experiencing decline in HD transmission but have improved survival and aging population [24]. The increasing burden among older adults observed in this study may, therefore, reflect both demographic changes and delayed detection of long-standing infections.

The proportion of childhood HD cases declined significantly during the study period. Despite the fact that reduction in number of leprosy cases among children alone may not be an indicator of reduction in active infection, childhood leprosy is widely regarded as an important indicator of recent infection [26]. The decline observed among individuals aged 0–14 years may, therefore, suggest reduction in active infection or improvements in early HD detection. Similar declines in childhood leprosy have been reported elsewhere in disease endemic countries which implemented strengthened surveillance and contact tracing strategies [26,27]. Any report of any childhood HD in a community indicates that transmission has not been completely interrupted, thus emphasizing the need for continued vigilance.

This study has also identified a significant increase in G1D at diagnosis, indicating that patients were diagnosed after nerve impairment had already occurred. Similar findings have been reported in other endemic settings, where stigma, limited community awareness, and inadequate access to diagnostic services have contributed to delayed diagnosis [13,28]. These findings highlight the need to strengthen early case detection and community-based interventions to facilitate timely diagnosis to prevent progression to severe disability.

The geographic distribution of cases also highlighted potential clustering of disease within certain locations, particularly the Ifakara division. This pattern may reflect localized transmission dynamics, higher population density, or reduced referrals to NLC. Health facility-based records often capture both epidemiological trends and healthcare-seeking behaviour, suggesting that strengthening community-level surveillance systems and decentralizing diagnostic services do improve early case detection and treatment.

The strengths of this study in analysis of HD over a 25-year period, provides a first-time long-term study on the epidemiological evolution of HD within a single referral center in Tanzania. The use of routinely collected clinical data allowed for detailed assessment of key indicators, including demographic characteristics, clinical classification, and disability status at diagnosis. Such longitudinal facility-based records provide valuable insights into temporal trends that may not be evident in short-term analyses.

However, some limitations should be taken into account when interpreting the findings. Firstly, this retrospective study relied on routinely collected NLC records, which are subject to incomplete documentation and missing data for certain variables such as type of patient, mode of detection, treatment outcome, and district of domicile. Secondly, given the study was conducted at a single specialized center, the findings may not fully represent the picture in the broader population and the observed epidemiological patterns may be influenced by referral patterns (referral bias) and late presentation. Thirdly, the cross-sectional design does not allow precise causal inference regarding the underlying drivers of the observed trends. Nonetheless, the study provides important evidence of the evolving epidemiology of HD in an endemic setting and highlights areas requiring strengthened management efforts.

## Conclusion

The findings of this study demonstrate a significant increase in the number of diagnosed HD cases, accompanied by a growing proportion of MB disease, increasing burden among older male adults, and rising rates of G1D at diagnosis. At the same time, the decline in childhood HD cases reflects there is progress in reduction of recent transmission. However, this finding alone cannot be taken as evidence of reduced recent transmission.

Strengthening community-based surveillance, improving awareness of early symptoms, enhancing timely diagnosis and treatment, expanding contact tracing, and integrating leprosy prevention strategies within primary healthcare systems may contribute to reducing HD burden. However, the effectiveness and feasibility of these approaches require further evaluation in different settings. Continued monitoring of epidemiological indicators at facility and community levels, and further research is needed to evaluate the effectiveness and feasibility of community-based surveillance, early symptom awareness and expanded contact tracing in different settings. Future studies should also examine how leprosy prevention and case detection can be effectively integrated into primary healthcare systems and assess their potential contribution to reducing delayed diagnosis and disability, and hence generate data and contribute to the National Tuberculosis and Leprosy Program towards elimination of the disease in Tanzania and elsewhere.

## Supporting information

S1 Data Data excel. (XLSX)
